# Perinatal outcomes associated with the use of glargine during pregnancy

**DOI:** 10.1111/j.1464-5491.2008.02485.x

**Published:** 2008-08

**Authors:** G Di Cianni, E Torlone, C Lencioni, M Bonomo, A Di Benedetto, A Napoli, E Vitacolonna, D Mannino, A Lapolla

**Affiliations:** Department of Endocrinology and Metabolism, Section of Diabetes and Metabolic Diseases, University of PisaPisa, Italy; * Department of Internal Medicine Endocrinology and Metabolism, University of PerugiaPerugia; ** Interdisciplinary Diabetes and Pregnancy Center, Niguarda Ca’ Granda HospitalMilano; † Department of Internal Medicine, University of MessinaMessina; ‡ Department of Clinical Sciences University La Sapienza RomeRoma; § Diabetes Unit, University of ChietiChieti; §§ Department of Endocrinology and Diabetology, Hospital ‘Bianchi, Malacrino, Morelli’Reggio Calabria; ¶ Department of Clinical and Surgical Sciences, University of PadovaPadova

**Keywords:** birthweight, congenital malformations, diabetes, insulin glargine, pregnancy

## Abstract

**Aims:**

Insulin glargine (IG), with its non-peaking action profile, might be useful in diabetic pregnancy. However, data on its safety are limited and its use during pregnancy is not recommended. This study focused on the effects of IG on perinatal outcome, particularly to estimate the rate of congenital anomalies and birthweight.

**Methods:**

This retrospective study included women with pre-gestational diabetes who used IG before (at least 1 month) and during pregnancy. For all women we recorded data regarding maternal glycaemic control and pregnancy outcome. We also compared women treated with IG throughout pregnancy and women who stopped taking IG at an earlier stage.

**Results:**

From 27 centres, 107 Type 1 diabetic pregnancies were identified. IG was started 10.3 ± 6.9 months before conception and in 57.4% of cases was stopped during the first trimester; 42.6% of women continued using it until the end of pregnancy. There were six abortions (four spontaneous and two induced) and five newborns (4.9%) with congenital anomalies. Glycaemic control, birthweight and the prevalence of macrosomia and neonatal morbidity were similar in women who used IG for the full term compared with those who stopped IG earlier during pregnancy.

**Conclusions:**

This study, although limited, suggests that IG is safe and effective; the rate of congenital malformations was within the range expected for diabetic pregnancies treated with more traditional forms of insulin. IG used throughout pregnancy did not seem to influence birthweight or increase adverse outcomes.

Diabet. Med. 25, 993–996 (2008)

## Introduction

Insulin glargine (IG), with its characteristic non-peaking action profile [1], could be useful in diabetic pregnancies, where strict glycaemic control and prevention of hypoglycaemia are essential to reduce adverse outcomes [2,3]. However, in view of the lack of controlled data on its safety, the use of IG is currently not recommended in pregnant women. Another potential concern is that the higher affinity of insulin glargine for the insulin-like growth factor 1 (IGF-1) receptor compared with other insulin preparations [4] might cause increased fetal growth, in spite of good glycaemic control. As a consequence, although IG is frequently prescribed for young patients with diabetes, its use during pregnancy is still limited and diabetologists have often discontinued IG during the periconceptional period or at first consultation during unplanned pregnancies.

To date, studies on IG in pregnancy have involved only a small number of women with pre-gestational diabetes [5–13] and more systematic studies are lacking. The Italian Diabetes Pregnancy Study Group has therefore promoted a national survey to collect data on the use of IG in women with pre-gestational diabetes. This study focuses on the effects of IG on the perinatal outcome, particularly examining the rate of congenital anomalies and birthweight.

## Methods

This retrospective study included 107 women from 27 Italian centres with pre-gestational diabetes who used IG before (at least 1 month) and during pregnancy. Centres were contacted by scientific diabetology or obstetric societies, during their national and regional meetings and via their websites. Investigators were asked to record data of all pregnancies in women with IG-treated pre-gestational diabetes that had ended before 31 December 2006. We collected data from women who had been treated with IG during their entire pregnancy and women who had stopped taking IG earlier in pregnancy. The study was approved by local ethics committees and women gave their informed consent to the collection of information from their medical records.

Basal clinical data were gathered from the first visit after conception: glycated haemoglobin (HbA_1c_), bodyweight and insulin therapy were recorded. HbA_1c_ values at the end of the pregnancy (1–2 weeks before delivery), frequency of episodes of severe hypoglycaemia and ketosis were used to assess overall glycaemic control during pregnancy. Severe hypoglycaemia was assumed in the event of hypoglycaemic emergencies where the woman required assistance. Ketosis was considered an episode of severe hyperglycaemia with an abnormally high concentration of ketone bodies in urine.

Regarding maternal outcome, gestational hypertension, pre-eclampsia, eclampsia, time and mode of delivery and maternal mortality were recorded. Information about abortions was obtained from hospital records. For neonatal outcome, data were collected for length and weight at birth, congenital malformations, shoulder dystocia, hypoglycaemia, fetal distress, jaundice, stillbirth and early neonatal mortality. Macrosomia was considered to be a birthweight > 4000 g; large-for-gestational age (LGA), if the baby's birthweight was > 90th percentile on the basis of the standard growth and development tables for the Italian population [14]. Neonatal ponderal index (PI) was defined as the ratio of weight to length cubed (g/cm^3^), with a PI > 2.85 g/cm^3^ considered as excessive growth [15]. Admissions to neonatal intensive care units (NICUs) were also recorded.

Neonatologists made the diagnosis of congenital anomalies and malformations were classified according to EUROCAT [16]. Fetal morbidity was classified according to the Obstetrical Quality indicator [17].

Results from women treated with IG throughout pregnancy were used to determine the effectiveness of IG on maternal glycaemic control and birthweight.

All statistical analyses were carried out using the SPSS statistical package for Windows (SPSS, Chicago, IL, USA). Discrete variables were compared using a χ^2^-test with Yates’ correction or Fisher's exact test, as appropriate. Continuous variables were compared using an unpaired two-sided *t*-test.

## Results

We identified 107 Type 1 diabetic pregnancies from the 27 centres. Pregnancy was unplanned in 94% of cases and folic acid supplementation was not used. All women started IG before conception (10.3 ± 6.9 months) as a basal–bolus regimen. The first diabetes assessment in pregnancy was at 8.5 ± 2.1 weeks of gestation; the average dose of IG was 19.2 ± 7.5 IU/daily (0.3 ± 0.1 IU/kg body weight). At this time, according to the policy adopted in each single centre, IG was stopped in 57.4% of cases, while the remaining 42.6% continued using it up to the end of pregnancy. Patients who stopped IG were switched to neutral protamine Hagedorn (NPH) insulin (usually in the morning and at bed-time), with the usual pre-meal insulin.

In all women, glycaemic control improved during pregnancy. There were no differences in clinical and metabolic parameters between women who stopped taking IG and those who continued (Table 1).

**Table 1:** Maternal and fetal outcome according to type of insulin treatment

	Group A (*n* = 43)	Group B (*n* = 58)	*P*
Mothers			
Age (years)	30.6 ± 3.5	30.4 ± 4.1	0.79
Diabetes duration (years)	16.8 ± 8.7	15.9 ± 6.5	0.55
Pre-pregnancy BMI (kg/m^2^)	23.2 ± 4.9	24.2 ± 3.4	0.325
Weight gain (kg)	14.1 ± 4.1	13.3 ± 4.4	0.35
HbA_1c_ (%)			
—first measurement in pregnancy	7.7 ± 1.32	7.6 ± 1.09	0.688
—at the end of pregnancy	6.5 ± 0.79	6.5 ± 0.91	0.97
Patients with hypoglycaemic episodes (%)	9.3	12.1	1.00
Patients with episodes of ketosis (%)	11.6	6.9	0.56
Gestational hypertension (%)	2.3	3.4	0.32
Pre-eclampsia (%)	2.3	8.6	0.8
Time of delivery (weeks)	37.2 ± 1.5	36.4 ± 2.4	0.045
Caesarean section (%)	79.1	79.3	0.89
Newborns			
Large for gestational age (%)	44.1	41.3	0.85
Ponderal index (kg/cm^3^)	2.8 ± 0.4	2.8 ± 0.3	0.92
Ponderal index > 2.85 (%)	45.0	46.9	1
Congenital malformations (%)	4.7	5.2	1
NICU admissions (%)	25.7	21.5	0.4
Neonatal hypoglycaemia (%)	14.6	17.2	0.72
Hyperbilirubinaemia (%)	19.5	22.2	0.62

Group A: patients with insulin glargine throughout pregnancy.

Group B: patients who stopped insulin glargine in pregnancy and started using intermediate human insulin.

BMI, body mass index; HbA_1c_, glycated haemoglobin; NICU, neonatal intensive care unit.

Six pregnancies were discontinued because of abortion—four spontaneous and two induced.

Delivery occurred in 101 pregnancies at 36.7 ± 2.1 weeks of gestation. The rates of pre-term deliveries and Caesarean section were 29.7 and 79.2% respectively. No maternal death was reported. All babies were living at birth; one baby, born preterm at the 29th week of gestation with a weight of 1215 g, died a few days after delivery; 23.3% of newborns were admitted to a NICU.

The rates of LGA and excessive growth (PI > 2.85 g/cm^3^) did not differ significantly between women who used IG throughout the pregnancy and those who stopped it earlier (Table 1). The rates of macrosomia were also similar in the two groups (20.9 and 14.8%, respectively). Five newborns had congenital malformations (4.95%), two cardiovascular, two genitourinary and one osteoarticular. Mothers of malformed infants were significantly older (*P* < 0.001) than the others and had had diabetes for longer (*P* < 0.04).

## Discussion

This survey collected data on 107 pregnancies of women with Type 1 diabetes. This is one of the most extensive surveys reported on this topic in recent years [5–13]. Almost all the pregnancies were unplanned; in view of the lack of controlled data on the safety and efficacy of IG, when pregnancy is planned diabetologists usually change the drug. For the same reason, as a policy of the diabetes centres, in our series IG was stopped at confirmation of pregnancy in a large number of women.

We therefore identified two groups of pregnant women (Table 1) who were clinically comparable; this was useful as a basis for assessing the effects of IG on maternal glycaemic control and on birthweight.

To determine the safety of IG in pregnancy, the study first focused on congenital malformation, given that IG was used during embryogenesis in all women. The rate of congenital anomalies was 4.95%. This figure is within the range (from 3.2 to 9%) expected for diabetic pregnancies treated with other forms of insulin, as recently reported in several European countries [18–23]. Moreover, in a previous multicentre survey of 504 Italian women with pre-gestational Type 1 diabetes (none taking IG either before or during pregnancy), we found a similar rate of congenital malformations of 5.9% [24]. It is worth noting that 19 out of 27 centres were involved in both surveys and that the two cohorts of pregnant diabetic women had similar clinical and metabolic characteristics. Thus, the present results suggest that IG has no negative effects on embryo–fetal development.

Another issue is the potential effect on fetal growth. One concern is that the higher affinity of IG for IGF-1 receptor compared with other insulin preparations [4] could potentially cause fetal macrosomia. However, in our series, the use of IG throughout pregnancy was not associated with higher birthweight or any change in neonatal outcome. In addition, the prevalence of macrosomia and/or LGA infants was no different from women who used NPH insulin in pregnancy. This result might possibly be because of comparable metabolic parameters (HbA_1c_ at the end of pregnancy, episodes of hypoglycaemia and ketosis) in the two groups, factors which are the main determinants of birthweight in diabetic pregnancies [25]. Thus, as recently reported in other studies [11,12], the absence of higher rates of macrosomic and LGA babies in women treated with IG does seem to exclude any increase in the risk of fetal overgrowth as a result of this type of therapy.

Although we are aware of the limited size of our cohort and the need for larger randomized trials in order to strengthen the findings, this survey, which at present is one of the largest reporting pregnancy outcomes in women treated with IG, suggests that IG is safe and effective. IG use did not increase the rate of congenital malformations in women with pre-gestational diabetes and did not seem to influence birthweight. In conclusion, IG in pregnancy seems to give much the same maternal–neonatal outcomes as older insulins.

### Participating investigators

The members of the Italian Diabetes and Pregnancy Study Group—Italian Society of Diabetology include: Graziano Di Cianni (Pisa), Elisabetta Torlone (Perugia) Cristina Lencioni (Pisa), Alessandra Battezzati (Alessandria), Matteo Bonomo (Milano), Daniela Bruttomesso (Padova), Gelsomina Capuano (Salerno), Silvana Caronna (Parma), Adolfo Ciavarella (Bologna), Maria Grazia Dalfrà (Padova), Eugenio De Feo (Napoli), Antonino Di Benedetto (Messina), Maria Dolci (Massa), Ivano Franzetti (Varese), Raffaella Fresa (Cava dei Tirreni-Salerno), Paola Gelisio (Mestre-Venezia), Alessandra Ghio (Pisa), Maria Pia Imbergamo (Palermo), Giovanna Losato (Rovigo), Valeria Manicardi (Reggio Emilia), Domenico Mannino (Reggio Calabria), Guido Menato (Torino), Marina Miola (Schio-Vicenza), Elena Mion (Milano), Mary Mori (Massa), Angela Napoli (Roma), Alessandro Pianta (Bassano Del Grappa-Treviso), Costanza Santini (Cesena), Laura Sciocca (Catania), Giancarlo Tonolo (Sassari), Laura Tonutti (Udine), Carla Tortul (Monfalcone-Gorizia), Ester Vitacolonna (Chieti), Laura Volpe (Pisa) and Annunziata Lapolla (Padova).

## Abbreviations

HbA_1c_: glycated haemoglobin
IFG-1: insulin-like growth factor 1
IG: insulin glargine
LGA: large for gestational age
NICU: neonatal intensive care unit
NPH: neutral protamine Hagedorn
PI: ponderal index
